# Hemispheric asymmetry for global-local processing: Effects of stimulus category and ageing

**DOI:** 10.1177/17470218241280800

**Published:** 2024-11-11

**Authors:** Haiwen Chen, Jolene A Cox, Anne M Aimola Davies

**Affiliations:** 1 School of Medicine and Psychology, The Australian National University, Canberra, ACT, Australia; 2 Centre for Human Factors and Sociotechnical Systems, University of the Sunshine Coast, Sippy Downs, QLD, Australia

**Keywords:** Global-local processing, hemispheric asymmetry, verbal-nonverbal processing, stimulus category, right-hemisphere ageing

## Abstract

Hemispheric asymmetry has been reported for global-local processing in young and older adults, with global processing specialised in the right hemisphere (RH-global specialisation) and local processing specialised in the left hemisphere (LH-local specialisation). Questions persist regarding the extent to which hemispheric asymmetry is influenced by stimulus category (verbal stimuli processed in the left hemisphere; visuospatial stimuli processed in the right hemisphere). Some evidence suggests stimulus category does not influence hemispheric asymmetry (stimulus-independent account) while other evidence suggests it does (stimulus-dependent account). In older adults, there is evidence of a local-processing advantage, believed to result from slower and less accurate performance in right-hemisphere compared to left-hemisphere functioning—the right-hemisphere ageing hypothesis. We examined hemispheric asymmetry for global-local processing in young and older participants with three hierarchical figures (letters, verbalisable objects, and nonverbalisable shapes), in a within-subjects design using a divided-attention paradigm and unilateral presentation. Our findings for letters and verbalisable objects support the stimulus-independent account—young and older participants demonstrated RH-global specialisation and LH-local specialisation regardless of stimulus category. In older participants, we also found a local-processing advantage for all three stimulus categories—an advantage best explained as faster and more accurate performance in local processing regardless of the visual field of stimulus presentation. Overall, we found hemispheric asymmetry for global-local processing in both young and older adults, and differences in global processing between young and older adults. Future investigation is suggested for the hemispheric asymmetry found in global-local processing of nonverbalisable shapes, and the mechanisms underlying age-related changes in global processing.

Our visual environment consists of hierarchical structures. For instance, a scene with many trees can be perceived as either an entire forest or as individual trees within the forest. To navigate in such a complex visual environment, our visual system relies on visual attentional mechanisms to process relevant information selectively (Carrasco, 2011). One such mechanism is global-local processing, which involves directing attention either globally (e.g., to the overall visual scene, such as the forest) or locally (e.g., to the finer elements within the scene, such as individual trees or even the leaves on a tree). Hemispheric asymmetry has been demonstrated for global-local processing (Yovel et al., 2001), with the right hemisphere (RH) specialised for global processing (i.e., RH-global specialisation) and the left hemisphere (LH) specialised for local processing (i.e., LH-local specialisation). Navon’s (1977) hierarchical figures, in which a large stimulus is made up of several smaller stimuli (e.g., a large global letter “U” is made up of smaller local letters “T”), has been widely used to study hemispheric asymmetry for global-local processing.

Hemispheric asymmetry for global-local processing has been demonstrated in neuropsychological studies, neuroimaging studies and behavioural studies. Neuropsychological studies have demonstrated that patients with lesions to their RH exhibit deficits in global processing, whereas patients with lesions to their LH exhibit deficits in local processing (e.g., Delis et al., 1986; Lamb et al., 1990; Robertson et al., 1988). Neuroimaging studies with healthy young adults further support the finding of hemispheric asymmetry for global-local processing by demonstrating greater RH activation for global processing and greater LH activation for local processing (e.g., Fink et al., 1996; Fink, Halligan et al., 1997; Martens & Hübner, 2013; Martinez et al., 1997). Behavioural studies, using a hierarchical-figures paradigm, within a divided visual field presentation, have provided additional support by demonstrating both reaction time (RT) and accuracy differences for global-local processing in the two hemispheres. For example, RT differences in hemispheric asymmetry were found by Hübner (1998), Sergent (1982), Van Kleeck (1989), and Yovel et al. (2001), and accuracy differences were found by Brederoo et al. (2017). The logic behind using the divided visual field presentation was that hierarchical stimuli briefly presented unilaterally to the left visual field (LVF) would be processed first by the RH, and hierarchical stimuli briefly presented unilaterally to the right visual field (RVF) would be processed first by the LH (Bourne, 2006). In these behavioural studies, RH-global specialisation was indicated by faster RT and/or higher accuracy for the global-level stimuli presented in the LVF/RH compared to the RVF/LH, and LH-local specialisation was indicated by faster RT and/or higher accuracy for the local-level stimuli presented in the RVF/LH compared to the LVF/RH.

## Effects of stimulus category

Previous research on RH-global specialisation and LH-local specialisation has primarily been based on hierarchical figures made up of letters (see Yovel et al., 2001). It has been suggested that the observed hemispheric asymmetry for global-local processing may be influenced by the stimulus category itself (Fink, Marshall, et al., 1997; Kéïta & Bedoin, 2011), as the LH is more specialised for verbal processing (e.g., letters) and the RH is more specialised for visuospatial processing (e.g., objects and shapes; see Dien, 2009). Specifically, the proposal is that the LH-local specialisation observed in studies using letters may have been driven by greater involvement of the LH in processing the letters (Fink, Marshall, et al., 1997). Five studies have addressed this hypothesis by directly comparing findings from hierarchical letters with those from hierarchical objects (see Table 1 for a summary), leading to two contrasting accounts: a *stimulus-independent* account and a *stimulus-dependent* account.

**Table 1.** Methodological differences in studies examining the effects of stimulus category.

	Study	Young participants N/Mean age	Measures	Stimulus category	Stimulus size (degree)		Local-level density	Stimulus presentation	Eccentricity (°from fixation)	Presentation duration	Task type	Response mode	Dependent variable	Hemispheric asymmetry findings	
					Global size	Local size								RH specialisation	LH specialisation
1	Johannes et al. (1996)	*N* = 13/26.7 years	Neuroimaging	Shapes	7.20° × 7.20°	1.16° × 1.16°	17	Central	NA	100 ms	Divided	Go/No go	ERP amplitude	—	—
2	Martinez et al. (1997)	*N* = 11/27 years	Behavioural	Shapes	2.30° × 3.60°	0.29° × 0.45°	16	Unilateral/Central	NR	50 ms	Focused	Go/No go	RT	—	**+** LH-local
			Neuroimaging	Shapes	2.30° × 3.60°	0.29° × 0.45°	16	Central	NA	50 ms	Focused	Mental counting^a^	Area of activation	**+** RH-global	**+** LH-local
3	Fink, Halligan, et al. (1997)	*N* = 10/19–35 years	Neuroimaging	Letters	10.00° × 24.00°	1.86° × 3.01°	11–18	Central	NA	300 ms	Focused	Naming	RBF	**+** RH-global	**+** LH-local
4	Fink, Marshall, et al. (1997)	*N* = 10/19–32 years	Neuroimaging	Objects	16.00° × 16.00°	1.00° × 1.00°	47–49	Central	NA	300 ms	Focused	Naming	RBF	**+** **RH-local**	**+** **LH-global**
5	Bedson and Turnbull (2002)	*N* = 15/27 years	Behavioural	Letters	NR	NR	13–17	Bilateral	NR	150 ms	Divided	Go/No go	Accuracy	**+** RH-global	—
			Behavioural	Objects	NR	NR	13–17	Bilateral	NR	150 ms	Divided	Go/No go	Accuracy	**+** RH-global	—
6	Kéïta & Bedoin (2011)	*N* = 32/22.7 years *N* = 32/22.5 years	Behavioural	Letters	3.80° × 4.00°	0.35° × 0.40°	16–26	Unilateral	2.00°	175 ms	Divided	Forced choice	RT/Accuracy	RT: **+** RH-global Accuracy: —	RT: — Accuracy: **+** LH-local
			Behavioural	Objects	3.80° × 4.00°	0.35° × 0.40°	24–32	Unilateral	2.00°	175 ms	Divided	Forced choice	RT/Accuracy	RT: — Accuracy: **+** **RH-local**	RT: — Accuracy: **+** **LH-global**
7	Brederoo et al. (2017)	*N* = 17/19.5 years	Behavioural	Letters	3.50° × 3.50°	0.49° × 0.49°	7–13	Unilateral/Bilateral	0.50°	120 ms	Divided	Forced choice	Accuracy	**+** RH-global	**+** LH-local
			Behavioural	Objects	3.50° × 3.50°	0.49° × 0.49°	8–12	Unilateral/Bilateral	0.50°	120 ms	Divided	Forced choice	Accuracy	**+** RH-global	**+** LH-local
8	Present Study (2024)	*N* = 27/20.59 years	Behavioural	Letters	2.30° × 3.08°	0.40° × 0.50°	7–10	Unilateral	0.50°	150 ms	Divided	Go/No go	RT/Accuracy	RT: **+** RH-global Accuracy: —	RT: **+** LH-local Accuracy: —
			Behavioural	Objects	3.30° × 3.30°	0.50° × 0.50°	10–15	Unilateral	0.50°	150 ms	Divided	Go/No go	RT/Accuracy	RT, accuracy: **+** RH-global	RT, accuracy: **+** LH-local
			Behavioural	Shapes	3.30° × 3.30°	0.50° × 0.50°	10–15	Unilateral	0.50°	150 ms	Divided	Go/No go	RT/Accuracy	—	—

The studies are listed in chronological order of publication year. ERP: event-related potentials; RBF: relative regional blood flow; NA: not applicable; NR: not reported; +: significant findings found for RH or LH specialisation; —: no significant findings found for RH or LH specialisation; RH-global: right hemisphere specialisation for global processing; LH-local: left hemisphere specialisation for local processing; RH-local: right hemisphere specialisation for local processing; LH-global: left hemisphere specialisation for global processing.

a Martinez et al.’s (1997) mental-counting response mode for their neuroimaging task required participants to keep a mental count of the number of times the target appeared at the attended processing level (global level *or* local level).

Supporters of the *stimulus-independent* account have argued that hemispheric asymmetry for global-local processing is not influenced by stimulus category. Brederoo et al. (2017) examined hemispheric asymmetry for global-local processing using hierarchical letters and hierarchical objects made up of geometric figures (e.g., crosses, rectangles, and diamonds). Their accuracy data demonstrated RH-global specialisation and LH-local specialisation for both letters and objects (also see Bedson & Turnbull, 2002). The *stimulus-independent* account is explained by the double-filtering by frequency theory (Ivry & Robertson, 1998), which posits that the selection of visual information occurs in two stages (Flevaris & Robertson, 2016), starting with the selection of a specific range of spatial frequencies from incoming spectra, and followed by processing of low spatial frequencies in the RH and high spatial frequencies in the LH (see also Sergent, 1982). Brederoo et al. further argued that the processing of low spatial frequencies in the RH results in RH-global specialisation and the processing of high spatial frequencies in the LH results in LH-local specialisation. This hemispheric asymmetry for global-local processing can be observed across different stimulus categories, as long as they have similar spatial frequency spectra. That is, the global level of a hierarchical figure, irrespective of stimulus category, is always specified by low spatial frequencies, and the local level is always specified by high spatial frequencies.

Supporters of the *stimulus-dependent* account have argued that stimulus categories with higher demands for visuospatial processing *reverse* the hemispheric asymmetry for global-local processing, resulting in RH-*local* specialisation and LH-*global* specialisation for objects. Two consecutive neuroimaging studies compared hierarchical letters (Fink, Halligan, et al., 1997) and hierarchical objects made up of familiar figures (e.g., hearts and stars; Fink, Marshall, et al., 1997). Behavioural data were not reported in these studies but the neuroimaging data revealed that, for letters, there was RH-global specialisation and LH-local specialisation; whereas, for objects, the pattern of hemispheric asymmetry was reversed, revealing RH-*local* specialisation and LH-*global* specialisation. Fink, Marshall, et al. (1997) proposed that this reversal of hemispheric specialisation could be explained by a top-down process that automatically assigns local processing to the hemisphere specialised for the verbal or visuospatial information involved in the stimulus while assigning global processing to the other hemisphere. Kéïta and Bedoin (2011) replicated these findings with behavioural data, by comparing participants’ RT and accuracy performance on hierarchical letters and hierarchical objects made up of familiar figures (e.g., hearts and stars). For letters, Kéïta and Bedoin demonstrated RH-global specialisation (as indicated by their RT data) and LH-local specialisation (as indicated by their accuracy data); whereas, for objects, only the accuracy data supported the reverse pattern of RH-*local* specialisation and LH-*global* specialisation.

The researchers (Brederoo et al., 2017; Fink, Halligan, et al., 1997; Fink, Marshall, et al., 1997; Kéïta & Bedoin, 2011) who have directly investigated the effects of stimulus category proposed that only visuospatial processing was required for their hierarchical objects, but it is worth noting that these hierarchical objects were made up of familiar verbalisable objects that could be associated with verbal labels. Only two studies (Johannes et al., 1996; Martinez et al., 1997) have investigated hemispheric asymmetry for global-local processing using novel nonverbalisable shapes, to which verbal labels could not easily be applied. Johannes et al. (1996) used electrophysiological measures and reported a LH advantage for global and local nonverbalisable shapes, thus not finding support for either the stimulus-independent or stimulus-dependent accounts. Martinez et al. (1997) used both behavioural and neuroimaging measures and found support for the stimulus-independent account in their neuroimaging data (i.e., RH activation for global nonverbalisable shapes and LH activation for local nonverbalisable shapes, with the strength of the RH-global activation greater than that of the LH-local activation). In their behavioural data, they reported LH-local specialisation for nonverbalisable shapes in the absence of RH-global specialisation. Neither study included a control stimulus category (i.e., neither letters nor verbalisable objects) when examining hemispheric asymmetry in global-local processing across different stimulus categories. Consequently, questions remain about the two theoretical accounts for the effect of stimulus category.

## Effects of ageing

Ageing is related to changes in many aspects of visual attention, including global-local processing. Widely reported behavioural findings have revealed that older adults demonstrate a local-processing advantage when compared to young adults. Specifically, older adults demonstrate faster RT or higher accuracy in detecting stimuli at the local level relative to the global level, both for centrally-presented stimuli (Insch et al., 2012; Lux et al., 2008; Slavin et al., 2002) and for unilaterally-presented stimuli (Oken et al., 1999).^1^

The right-hemisphere ageing hypothesis (Albert, 1981; Brown & Jaffe, 1975; Klisz, 1978) has been put forth to explain this age-related local-processing advantage. The main idea here is that the RH is more vulnerable to ageing than the LH, with LH functions remaining relatively stable across the lifespan (see Goldstein & Shelly, 1981). Additional support for the right-hemisphere ageing hypothesis includes the finding that older adults have a rightward attentional bias in the landmark task, with the right half of lines perceived as being longer than the left half of lines (Schmitz & Peigneux, 2011), and that older adults have greater difficulties with: visuospatial working memory compared to verbal working memory (Jenkins et al., 2000); low-spatial-frequency information compared to high-spatial-frequency information (Wang et al., 2019); and reorienting attention from invalid cues in the RVF to targets in the LVF in a spatial-cueing task (Guilbert et al., 2019).

There are four outstanding questions that require further investigation of the extent to which the right-hemisphere ageing hypothesis explains the local-processing advantage found in older adults:

1. Why are there inconsistent findings for the local-processing advantage in older adults, which also sometimes include findings for a global-processing advantage?
2. Is the local-processing advantage found in older adults driven by age-related changes in local processing or age-related changes in global processing?
3. If the local-processing advantage in older adults is driven by age-related difficulties with global processing, can these difficulties be attributed to age-related changes in RH functioning?
4. Does stimulus category (letters, verbalisable objects, and nonverbalisable shapes) affect the local-processing advantage?

To address the first question, the difference in the size and number of local-level stimuli in the hierarchical figures used in previous studies needs to be considered (see Table 2 for a summary). For example, a local-processing advantage (i.e., local-level stimuli detected faster or more accurately than global-level stimuli) was found in older adults in the study by Oken et al. (1999), in which nine to 12 local-level stimuli were used in each hierarchical figure. In contrast, a global-processing advantage (i.e., global-level stimuli detected faster or more accurately than local-level stimuli) was found in older adults in studies that used a larger number of local-level stimuli.^2^ The global-processing advantage was found in RT differences, by Agnew et al. (2016; 18 to 22 local-level stimuli), Georgiou-Karistianis et al. (2006; 14 to 22 local-level stimuli), and Roux and Ceccaldi (2001; 18 to 22 local-level stimuli), and in accuracy differences, by Bruyer et al. (2003; 11 to 17 local-level stimuli), and Roux and Ceccaldi (2001; 18 to 22 local-level stimuli). It has been posited that when a larger number of local-level stimuli are used to make up a hierarchical figure, the global-level stimulus becomes more perceptually salient than the local-level stimuli (see Yovel et al., 2001). Consequently, detection of the global-level stimulus may have been made easier in these studies, resulting in the finding of a global-processing advantage in older adults.

**Table 2.** Methodological differences in studies examining the effects of ageing.

	Study	Participants N/Mean age		Stimulus category	Stimulus size (degree)		Local-level density	Stimulus presentation	Eccentricity (°from fixation)	Presentation duration	Task type	Response mode	Dependent variable	Processing advantage findings	
		Young	Older		Global size	Local size								Young	Older
1	Oken et al. (1999)	*N* = 20/23.4 years	*N* = 25/83.6 years	Objects	12.00°	1.00°–1.50°	9–12	Unilateral	8.00°	100 ms	Focused	Go/No-go	RT	Equal	**Local**
													Accuracy	Equal	Equal
2	Bruyer & Scailquin (2000)	*N* = 16/19.25 years	*N* = 16/68.69 years	Letters	3.11° × 3.11°	0.39° × 0.39°	NR	Quadrants^a^	3.24°	50 ms	Focused	Forced choice	RT	Global	Global
													Accuracy	Global	Global
		*N* = 16/19.44 years	*N* = 21/69.37 years	Letters	3.11° × 3.11°	0.39° × 0.39°	NR	Quadrants^a^	3.24°	50 ms	Focused	Forced choice	RT	Global	Global
													Accuracy	Equal	Global
		*N* = 16/19.17 years	*N* = 21/72.60 years	Letters	3.11° × 3.11°	0.39° × 0.39°	NR	Quadrants^a^	3.24°	Young: 50 ms Older: 200 ms	Focused	Forced choice	RT	Global	Global
													Accuracy	Global	Global
3	Roux & Ceccaldi (2001)	*N* = 16/22 years	*N* = 21/70 years	Letters	2.80° × 4.00°	0.30° × 0.50°	18–22	Quadrants^a^	2.80°	100 ms	Focused	Forced choice	RT	Global	Global
													Accuracy	Global	Global
4	Slavin et al. (2002)	*N* = 12/26.9 years	*N* = 12/75.8 years	Numbers	1.60°–3.55° × 6.75°	0.34° × 0.57°	16–21	Central	NA	No limit	Focused	Forced choice	RT	Global	**Local**
													Accuracy	Global	NR
5	Bruyer et al. (2003)	*N* = 16/21.25 years	*N* = 16/70.69 years	Letters	3.11° × 3.11°	0.39° × 0.39°	11–17	Quadrants^a^	3.24°	50 ms	Focused	Forced choice	RT	Global	Global
													Accuracy	Global	Global
		*N* = 16/20.19 years	*N* = 16/69.44 years	Letters	3.11° × 3.11°	0.39° × 0.39°	11–17	Quadrants^a^	3.24°	50 ms	Focused	Forced choice	RT	Global	Global
													Accuracy	Global	Global
6	Georgiou-Karistianis et al. (2006)	*N* = 20/28.10 years	*N* = 20/70.35 years	Numbers	1.24°–2.86° × 5.33°	0.19°–0.28° × 0.38°	14–22	Central	NA	No limit	Focused	Forced choice	RT	Global	Global
													Accuracy	Equal	Equal
7	Lux et al. (2008)	*N* = 22/21.7 years	*N* = 22/58.4 years	Letters	5.70° × 8.50°	0.80° × 1.00°	5 × 7	Central	NA	150 ms	Focused	Forced choice	RT	Equal	**Local**
													Accuracy	Equal	Equal
				Letters	5.70° × 8.50°	0.80° × 1.00°	5 × 7	Central	NA	150 ms	Divided	Forced choice	RT	Equal	**Local**
													Accuracy	Equal	**Local**
8	Staudinger et al. (2011)	*N* = 20/22.1 years	*N* = 20/57.0 years	Letters	5.70° × 8.50°	0.80° × 1.00°	3 × 5, 4 × 6, 5 × 7, 6 × 8, 7 × 9	Central	NA	150 ms	Focused	Forced choice	RT	Global	Equal
													Accuracy	Global	Global
9	Insch et al. (2012)	*N* = 52/25.81 years	*N* = 34/73.56 years	Objects	8.00°	0.75°	NR	Central	NA	NR	Focused	Forced choice	Accuracy	Equal	**Local**
10	Agnew et al. (2016)	*N* = 17/19.9 years	*N* = 15/65.4 years	Letters	3.00° × 4.00°	0.30° × 0.50°	18–22	Central	NA	No limit	Focused	Forced choice	RT	Global	Global
													Accuracy	Local	**Local**
11	Lithfous et al. (2016)	*N* = 22/24.43 years	*N* = 22/69.14 years	Letters	Sparse: 13.00° × 13.00° Dense: 4.50° × 4.50°	Sparse: NR Dense: NR	Sparse: 12–13 Dense: 49	Central	NA	100 ms	Focused	Forced choice	RT	Sparse: Equal Dense: Global	Sparse: Equal Dense: Equal
													Accuracy	Sparse: Equal Dense: Global	Sparse: **Local** Dense: Equal
12	Present Study (2024)	*N* = 27/20.59 years	*N* = 34/72.23 years	Letters	2.30° × 3.08°	0.40° × 0.50°	7–10	Unilateral	0.50°	150 ms	Divided	Go/No go	RT/accuracy	RT: Local Accuracy: Equal	RT, accuracy: **Local**
		*N* = 27/20.59 years	*N* = 32/71.24 years	Objects	3.30° × 3.30°	0.50° × 0.50°	10–15	Unilateral	0.50°	150 ms	Divided	Go/No go	RT/accuracy	RT: Equal Accuracy: Equal	RT, accuracy: **Local**
		*N* = 27/20.59 years	*N* = 30/70.94 years	Shapes	3.30° × 3.30°	0.50° × 0.50°	10–15	Unilateral	0.50°	150 ms	Divided	Go/No go	RT/accuracy	RT: Equal Accuracy: Equal	RT, accuracy: **Local**

The studies are listed in chronological order of publication year. NA: not applicable; NR: not reported; Local: a local-processing advantage (i.e., significantly faster RT or higher accuracy for detecting local-level stimuli compared to global-level stimuli); Global: a global-processing advantage (i.e., significantly faster RT or higher accuracy for detecting global-level stimuli compared to local-level stimuli); —: no significant findings for a local-processing advantage in older participants; Equal: equal salience (i.e., no significant RT or accuracy differences in detecting global-level stimuli and local-level stimuli); Bold highlighting indicates the research findings for a local-processing advantage in older adults.

a Bruyer and Scailquin (2000), Roux and Ceccaldi (2001), and Bruyer et al. (2003) presented their stimuli in quadrants (i.e., stimuli were presented in one of the four quadrants around central fixation—upper LVF, lower LVF, upper RVF, lower RVF).

To address the second question, a between-group comparison needs to be incorporated into the statistical analysis of the findings. The local-processing advantage in older adults reported in previous studies was found by comparing the performance of older adults for global-level stimuli with that for local-level stimuli (Insch et al., 2012; Lux et al., 2008; Oken et al., 1999; Slavin et al., 2002). However, these studies did not report the performance differences in global-local processing for older adults compared to young adults. Consequently, the question remains as to whether the observed local-processing advantage stemmed from older adults demonstrating better performance in detecting local-level stimuli compared to young adults, or from older adults demonstrating worse performance in detecting global-level stimuli compared to young adults.

To address the third question, a divided visual field presentation needs to be incorporated into the experiment design. Although some studies have reported evidence for a local-processing advantage in the behavioural data (RT and accuracy) of older adults (Insch et al., 2012; Lux et al., 2008; Oken et al., 1999; Slavin et al., 2002), only Oken et al. presented stimuli unilaterally (i.e., presented stimuli either to the LVF or to the RVF) in a divided visual field presentation. Other studies have presented stimuli centrally, which is considered not to be an effective way to examine directly the effects of ageing on RH functioning and LH functioning (see Bourne, 2006 for a review of using a divided visual field presentation to examine hemispheric asymmetry). Consequently, although these studies reported a local-processing advantage in older adults, it is unclear whether this can be attributed to age-related changes in RH functioning. To the best of our knowledge, there are only four studies in the literature (Bruyer et al., 2003; Bruyer & Scailquin, 2000; Oken et al., 1999; Roux & Ceccaldi, 2001) that have adopted a divided visual field presentation in older adults. Of these four studies, only Oken et al. reported findings in regard to hemispheric asymmetry. Contrary to the right-hemisphere ageing hypothesis, Oken et al.’s RT results for older participants did not demonstrate age-related difficulties in overall performance in the LVF/RH compared to the RVF/LH (regardless of global-local processing level). Importantly, in addition to finding hemispheric asymmetry (i.e., global-level verbalisable objects detected faster in the LVF/RH compared to the RVF/LH, and local-level verbalisable objects detected faster in the RVF/LH compared to the LVF/RH), Oken et al. also reported a local-processing advantage—older participants detected local-level verbalisable objects faster than global-level verbalisable objects (regardless of visual field of presentation). One potential confounding factor in the Oken et al. findings of hemispheric asymmetry remains to be investigated, which is that their stimuli were presented at a large eccentricity of 8.00° from central fixation. It has been suggested that the RH is specialised for stimuli of low visual acuity (Christman, 1989; Sergent, 1983). Since visual acuity decreases as eccentricity increases from central fixation (Lindell & Nicholls, 2003), low visual acuity at this eccentricity may have inadvertently enhanced RH functioning, resulting in the intact overall performance in the LVF/RH observed in Oken et al.’s older participants.

To address the fourth question, a within-subjects comparison with more than one stimulus category needs to be incorporated into the experiment design. The local-processing advantage found in previous studies was based on participants tested with only one stimulus category, without a within-subjects comparison to other stimulus categories (e.g., letters only by Lux et al., 2008; numbers only by Slavin et al., 2002; verbalisable objects only by Oken et al., 1999). The question that arises is whether older adults will demonstrate the local-processing advantage for stimulus categories when there are different demands for verbal *versus* visuospatial processing.

## The present study

In the present study, we used a divided-attention paradigm, in which participants attended to both processing levels (i.e., global level and local level) at the same time, and a divided visual field presentation, in which hierarchical figures were presented unilaterally (i.e., presented either to the LVF or to the RVF) in a within-subjects experiment design. The stimuli were Navon’s (1977) hierarchical figures, and there were three stimulus categories: hierarchical letters (verbalisable), hierarchical objects (verbalisable), and hierarchical shapes (nonverbalisable). The hierarchical letters were composed of letters (e.g., the letter, S), which were meant to be strictly processed by their verbal labels. The hierarchical objects were composed of familiar figures (e.g., 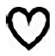; heart), to which verbal labels could easily be applied. The hierarchical shapes were composed of unfamiliar shape patterns (e.g., 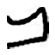), to which verbal labels could not easily be applied. All stimuli were presented at an eccentricity of 0.50° from central fixation.

This experiment setup allowed us to investigate two competing accounts of hemispheric asymmetry in global-local processing—the stimulus-independent account and the stimulus-dependent account. We aimed to examine systematically the effects of three different stimulus categories (letters, verbalisable objects, and nonverbalisable shapes) on hemispheric asymmetry for global-local processing in both young and older participants. Previous studies examining the effects of stimulus category on hemispheric asymmetry for global-local processing in young adults have only compared the findings for letter stimuli with those for verbalisable objects (e.g., Brederoo et al., 2017; Fink, Marshall, et al., 1997; Kéïta & Bedoin, 2011). No study thus far has explored the stimulus category of nonverbalisable shapes and compared these findings with those for letters or verbalisable objects in a within-subjects design.

This experiment setup also allowed us to investigate the extent to which the right-hemisphere ageing hypothesis (i.e., the RH is more vulnerable to ageing than the LH) can explain previous findings of a local-processing advantage in older adults. The four outstanding questions listed above were taken into account by: (1) setting the size of the hierarchical figure and the number of local-level stimuli to match closely with what Yovel et al. (2001) recommended for an equal salience design; (2) including a between-group comparison in the statistical analysis, thus for the first time providing a comparison of older adults’ performance for global processing and for local processing with that of young adults; (3a) using a divided visual field presentation, which is considered the best method for comparing left visual field/right-hemisphere performance with right visual field/left-hemisphere performance; (3b) using a small eccentricity of 0.50° from central fixation for unilateral stimulus presentation, as suggested by Yovel et al.; (4) conducting a within-subjects experiment including three stimulus categories, given that the age-related local-processing advantage found in previous studies was based on participants tested with only one stimulus category.

## Method

### Participants

A total of 63 participants were recruited for this study: 27 young participants were recruited from the student population at the Australian National University, and 36 older participants were recruited from the University of the Third Age and the broader community in Canberra. All participants self-reported being of European descent, and living in Australia. Participants were excluded from the statistical analyses if they did not meet the following five inclusion criteria:

- Right-handed, based on the Edinburgh Handedness Inventory (Oldfield, 1971). Participants were considered to be right-handed if their laterality scores were higher than + 50. One older participant scored lower than this laterality score cut-off and was excluded from all of the statistical analyses.
- Normal or corrected-to-normal vision, as indicated by in-laboratory testing with a Snellen chart (Snellen, 1868). No young or older participants were excluded.
- No history of self-reported neurological impairments. No young or older participants were excluded.
- No evidence of cognitive impairment, as assessed by the Montreal Cognitive Assessment (MoCA; Nasreddine et al., 2005), with a score of 24 or greater indicating intact cognitive functioning (see Pugh et al., 2018; Thomann et al., 2020). One older participant scored lower than this MoCA cut-off score of 24 and was excluded from all of the statistical analyses.
- High overall accuracy on *target-absent trials* (i.e., the hierarchical figures in which no target was presented, and therefore no response was required). A response to a target-absent trial indicated that the participant either did not discriminate targets from distractors or tended to respond quickly but with low accuracy. Different accuracy cut-offs were adopted based on the relative overall accuracy for the target-absent trials for the three stimulus categories (letters: 80% or greater; verbalisable objects: 70% or greater; and nonverbalisable shapes: 60% or greater). Two older participants, who did not meet the accuracy cut-off, were excluded from the statistical analyses for verbalisable objects and nonverbalisable shapes (but not from the analyses for letters), and two older participants, who did not meet the accuracy cut-off, were excluded from the statistical analyses for nonverbalisable shapes (but not from the analyses for letters or verbalisable objects).

In summary, of the 63 participants recruited for the present study, 27 young participants were included in all of the statistical analyses and two older participants were excluded from all of the statistical analyses. The final statistical analyses included a sample of 27 young participants and 34 older participants. Of these 34 older participants, four were excluded from some but not all of the statistical analyses. See Table 3 for full details. The power analysis conducted using G*Power (version 3.1) indicated that to achieve 85% power with α = .05, a minimal sample size of 23 young participants and 29 older participants was required.^3^ All participants provided written informed consent prior to participation in the study and received monetary compensation (or course credit for eligible young participants). The study was approved by the Australian National University Human Research Ethics Committee (Protocol: 2019/795).

**Table 3.** Demographic information and accuracy of target-absent trials for the young and older participants included in the statistical analyses for each of three stimulus categories (letters, verbalisable objects, and nonverbalisable shapes).

	Letters		Verbalisable objects		Nonverbalisable shapes	
	Young	Older	Young	Older	Young	Older
*N*	27	34	27	32	27	30
Female (*n*)	16	26	16	24	16	22
Female (%)	59.26%	76.47%	59.26%	75.00%	59.26%	73.33%
Age						
* M*	20.59	72.23	20.59	71.24	20.59	70.94
* SD*	2.45	8.02	2.45	7.98	2.45	8.08
* Range*	19–30	55–88	19–30	55–88	19–30	55–88
Accuracy for target-absent trials	97.24%	94.03%	96.18%	87.34%	96.30%	83.23%

### Apparatus and stimuli

Experiments were programmed in Inquisit (version 6.2.1) and were presented on a 27-inch Dell S2719DGF monitor with 60 Hz refresh rate and 2,560 × 1,440 pixels resolution. Participants’ RT and accuracy were recorded with a Cedrus RB-844 response pad. A forehead-and-chin rest was used to maintain a 57 cm constant eye-to-screen viewing distance for each participant.

The present study consisted of three experiments with hierarchical figures: hierarchical letters, hierarchical objects, and hierarchical shapes. The hierarchical letters were large global letters (size = 2.30° × 3.08°) composed of seven to 10 small local letters (size = 0.40° × 0.50°). The global and local size of the hierarchical letters was based on the equal salience experiment design reported by Yovel et al. (2001). The hierarchical objects were large global verbalisable objects (size = 3.30° × 3.30°) composed of 10 to 15 small local verbalisable objects (size = 0.50° × 0.50°). The hierarchical shapes were large global nonverbalisable shapes (size = 3.30° × 3.30°) composed of 10 to 15 small local nonverbalisable shapes (size = 0.50° × 0.50°). Although the structure of the verbalisable objects and nonverbalisable shapes was of the same visual complexity, both the verbalisable objects and the nonverbalisable shapes were visually more complex than the letters. Therefore, to achieve equal discriminability with the hierarchical letters at both the global and the local level, more local stimuli (i.e., 10 to 15 local stimuli for objects and shapes as compared to 7 to 10 local stimuli for letters) were used to make up the hierarchical verbalisable objects and the hierarchical nonverbalisable shapes. The average centre-to-centre distance between each pair of consecutive local stimuli for hierarchical letters was 0.73° (range: 0.60°–0.84°), for hierarchical verbalisable objects was 0.68° (range: 0.60°–0.80°), and for hierarchical nonverbalisable shapes was 0.73° (range: 0.66°–0.88°).

**Figure 1.**
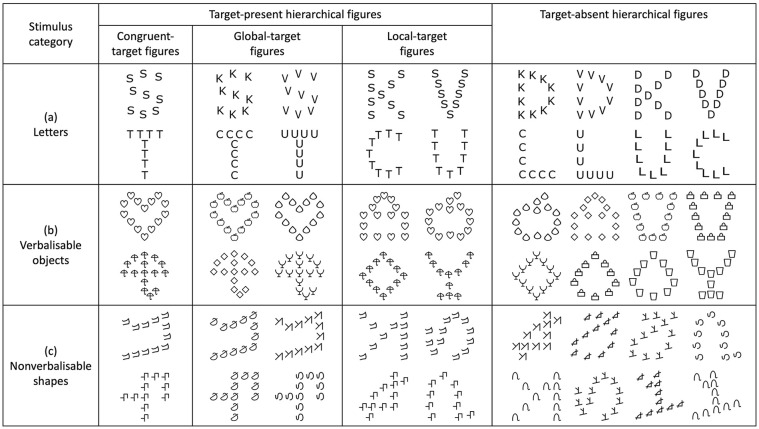
Hierarchical figures for three experiments: hierarchical letters, hierarchical verbalisable objects, and hierarchical nonverbalisable shapes. Panel (a) hierarchical-letters experiment: target letters were S and T, and distractor letters were D, C, K, L, U, and V. Panel (b) hierarchical verbalisable objects experiment: target objects were 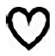 (heart) and 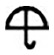 (umbrella), and distractor objects were 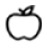 (apple), 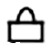 (briefcase), 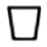 (cup), 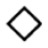
(diamond), 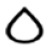 (drop), and 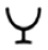 (glass). Panel (c) hierarchical nonverbalisable shapes experiment: target shapes were 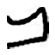 and 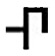, and distractor shapes were 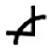, 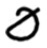, 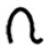, 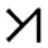, 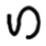, and 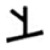.

For each of the three stimulus categories tested (letters, verbalisable objects, and nonverbalisable shapes), there were two targets and six distractors. The target letters were “S” and “T”. The target verbalisable objects were 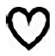 (heart) and 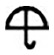 (umbrella). The target nonverbalisable shapes were 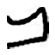 and 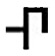. All of the other letters, verbalisable objects, and nonverbalisable shapes were distractors. Within a hierarchical figure, targets were either present (target-present hierarchical figures) or absent (target-absent hierarchical figures). There were three types of target-present hierarchical figures: congruent-target figures (the same target appeared at both the global and local level); global-target figures (one target appeared at the global level and one distractor appeared at the local level); and local-target figures (one target appeared at the local level and one distractor appeared at the global level). There was only one type of target-absent hierarchical figure, which was made up of two different distractors (one distractor appeared at the global level and one distractor appeared at the local level). There were 18 hierarchical figures for each of the three stimulus categories (18 hierarchical letters, 18 hierarchical verbalisable objects, 18 hierarchical nonverbalisable shapes), and for each stimulus category there were two congruent-target figures, four global-target figures, four local-target figures, and eight target-absent figures. See Figure 1 for full details.

The hierarchical figures were presented in black against a white background. For each trial, one hierarchical figure was presented unilaterally either to the LVF or to the RVF. The medial edge of the hierarchical figure was positioned 0.50° to the left or to the right of a central fixation cross (size = 0.40° × 0.40°).^4^ To prevent afterimages, two post-stimulus masks were presented (bilaterally) immediately after the presentation of a hierarchical figure. The medial edges of each of the post-stimulus masks were positioned 0.50° to the left or to the right of the central fixation cross. The post-stimulus mask comprised Gaussian white noise (300 pixels × 300 pixels), made up of noise elements with a Gaussian mean of 0 and standard deviation of 1. There were two sizes for the post-stimulus masks: 2.30° × 3.08° for hierarchical letters; 3.30° × 3.30° for hierarchical verbalisable objects and nonverbalisable shapes.

### Procedure

Participants were tested individually in a distraction-free room. To reduce the likelihood that participants would adopt verbal strategies for processing the verbalisable objects and nonverbalisable shapes, the order of the three experiments was not randomised.^5^ Given that nonverbalisable shapes were meant to be processed visuospatially, the hierarchical nonverbalisable shapes experiment was conducted first, followed by the hierarchical verbalisable objects experiment. The hierarchical-letters experiment was conducted last since letters were expected to be processed verbally. At the beginning of each of the three experiments (letters, verbalisable objects, and nonverbalisable shapes), participants were first shown all of the hierarchical figures to be used in that specific experiment, and then they performed two practice blocks to ensure both understanding and compliance with the task instructions for the experiment block. Feedback regarding accuracy was provided only in the practice blocks.

The experiment block for each of the three experiments (letters, verbalisable objects, and nonverbalisable shapes) consisted of 160 trials, presented in a random order. Of the 160 trials, 96 were target-present trials (consisting of 32 congruent-target trials, 32 global-target trials, and 32 local-target trials) and 64 were target-absent trials. Hierarchical figures were randomly presented either to the LVF or to the RVF in equal proportion: in 50% of trials, the hierarchical figures were presented to the LVF and in the remaining 50% of trials, the hierarchical figures were presented to the RVF. A divided-attention paradigm was adopted, in which participants were instructed to attend to both the global level and the local level simultaneously. A divided-attention paradigm has been suggested to produce a greater effect size for hemispheric asymmetry for global-local processing (Yovel et al., 2001). Participants were instructed to respond as quickly and as accurately as possible, by pressing a green-coloured button on the Cedrus response pad with their right index finger, when they saw a target appear at either the global level or the local level. They were instructed not to respond when a target did not appear at either the global level or the local level.

Each trial began with a central fixation cross (500 ms), followed by a hierarchical figure presented unilaterally either to the LVF or to the RVF (150 ms), and then two post-stimulus masks were presented bilaterally (1,000 ms) before the inter-trial interval (1,000 ms). No feedback was provided in the experiment trials. The presentation duration of 150 ms for the hierarchical figures was chosen because a short stimulus duration prevents eye movements away from central fixation and towards the stimulus. For this reason, previous researchers (Blanca et al., 1994; Bourne, 2006; Hunter & Brysbaert, 2008) have recommended a presentation duration range of between 50 ms and 180 ms, but no less than 50 ms because too short a presentation duration decreases visual acuity and can lead to enhanced RH activity (Boles & Karner, 1996; Evert & Kmen, 2003).

### Statistical analysis plan

Both RT and accuracy were analysed.^6^ For RT, gamma mixed-effects regression was adopted, which used RT data by trials instead of mean RT. The gamma distribution is positively skewed by nature and takes values between [0, +∞], which is better suited for capturing the properties of RT (see Lo & Andrews, 2015). For accuracy, logistic mixed-effects regression was adopted, which used accuracy data by trials (i.e., correct, incorrect) instead of mean accuracy (i.e., proportion of correct responses).

An omnibus analysis was conducted. The fixed effects included three within-subjects predictors: Level (global, local), Visual Field (LVF, RVF), and Stimulus Category (letters, verbalisable objects, and nonverbalisable shapes), and one between-subjects predictor: Age Group (young, older). First, the omnibus analysis explored the main effects and interactions for all four fixed effects. Second, the omnibus analysis examined overall performance differences for the three stimulus categories in young and older participants; these differences would be indicated by a significant Stimulus Category × Age Group interaction.

Three follow-up analyses were conducted for each of the three stimulus categories (letters, verbalisable objects, and nonverbalisable shapes). The fixed effects included two within-subjects predictors: Level (global, local) and Visual Field (LVF, RVF), and one between-subjects predictor: Age Group (young, older). The first aim of these follow-up analyses was to examine the research question regarding hemispheric asymmetry for global-local processing. Specifically, these follow-up analyses examined age-group differences in hemispheric asymmetry for global-local processing for each of the three stimulus categories (letters, verbalisable objects, and nonverbalisable shapes). Hemispheric asymmetry for global-local processing would be indicated by two contrasts in the post hoc pairwise comparisons for a significant Level × Visual Field interaction. RH-global specialisation would be indicated by significantly better performance in detecting global-level stimuli presented in the LVF/RH compared to the RVF/LH, and LH-local specialisation would be indicated by significantly better performance in detecting local-level stimuli presented in the RVF/LH compared to the LVF/RH (Brederoo et al., 2017; Hübner, 1998; Martin, 1979; Sergent, 1982; Van Kleeck, 1989; Yovel et al., 2001). Significant three-way interactions for Level (global, local) × Visual Field (LVF, RVF) × Age Group (young, older) were followed up with additional analyses within each of the two age groups (young, older). The fixed effects included two within-subjects predictors: Level (global, local) and Visual Field (LVF, RVF). The second aim of these follow-up analyses was to examine the research question regarding the age-related local-processing advantage in older participants, which would be indicated by a significant two-way interaction for Level (global, local) × Age Group (young, older). For all analyses, the dependent variable was RT for correct trials or accuracy for all trials, for each participant.

Participants were entered as random intercepts for all analyses. While it has been recommended that all within-subjects factors be included as random slopes to capture dependencies in the repeated-measures design (Barr, 2013), we did not include all within-subjects factors in some of the analyses to avoid over-fitting due to singular fits (i.e., the random slopes were too complex to be supported by the data). For the omnibus analysis, Level (global, local), Visual Field (LVF, RVF), and Stimulus Category (letters, verbalisable objects, and nonverbalisable shapes) were entered into the random slopes for accuracy, while only Level (global, local) and Visual Field (LVF, RVF) were entered into the random slopes for RT. For the follow-up analyses examining hemispheric asymmetry and the age-related local-processing advantage in each of the three stimulus categories (letters, verbalisable objects, and nonverbalisable shapes), Level (global, local) and Visual Field (LVF, RVF) were entered into the random slopes for RT, while only Level (global, local) was entered into the random slopes for accuracy. For the additional analyses examining hemispheric asymmetry within each of the two age groups (young, older), only Level (global, local) was entered into the random slopes for RT and accuracy to avoid over-fitting due to singular fits.

All analyses were carried out in *R* (version 4.0.5), and all the fixed effects were deviation-coded. The gamma mixed-effects regression with the identity link function and the logistic mixed-effects regression with the logit link function was conducted using the *“glmer”* function in the *“lme4”* package. The Type-III analyses of variance (ANOVA) tables with Wald chi-squares tests were obtained using the “*Anova*” function in the “*car*” package. Post hoc pairwise comparisons with Tukey-corrected *p* values were conducted using the “*emmeans*” function in the “*emmeans*” package. Estimated marginal means and standard errors were obtained using the “*effect*” function in the “*effects*” package. Post hoc pairwise comparisons report the mean difference (*MD*) in milliseconds for the RT data and the *MD* in log-odds-ratio scale for the accuracy data. Effect size *d* was computed for post hoc pairwise comparisons by dividing *MD* by the square root of the sum of variance for random intercepts, random slopes, and residuals (Westfall et al., 2014; also see Brysbaert & Stevens, 2018).^7^

## Results

### Omnibus analysis

The omnibus analysis indicated there were significant three-way and/or four-way interactions with Stimulus Category (letters, verbalisable objects, and nonverbalisable shapes) for RT and accuracy (see Table 4 for the main effects and interactions for RT and accuracy in the omnibus analysis). As planned, three follow-up analyses for each of the three stimulus categories were conducted to investigate the two main research questions regarding hemispheric asymmetry for global-local processing and the age-related local-processing advantage.

**Table 4.** Main effects and interactions of the omnibus analysis for reaction time and accuracy.

	Reaction Time			Accuracy		
	*χ* ^2^	*df*	*p*	*χ* ^2^	*df*	*p*
Level	194.42	1	<.001***	7.46	1	.006**
Visual Field	1.61	1	.20	0.003	1	.96
Stimulus Category	870.95	2	<.001***	97.25	2	<.001***
Age Group	162.58	1	<.001***	66.60	1	<.001***
Level × Visual Field	104.44	1	<.001***	18.67	1	<.001***
Level × Stimulus Category	601.16	2	<.001***	2.54	2	.28
Level × Age Group	620.58	1	<.001***	36.68	1	<.001***
Visual Field × Stimulus Category	1.69	2	.43	1.67	2	.43
Visual Field × Age Group	0.85	1	.36	0.07	1	.79
Stimulus Category × Age Group	953.23	2	<.001***	11.62	2	.003**
Level × Visual Field × Stimulus Category	67.51	2	<.001***	5.31	2	.07
Level × Visual Field × Age Group	52.94	1	<.001***	6.18	1	.01*
Level × Stimulus Category × Age Group	467.53	2	<.001***	17.46	2	<.001***
Visual Field × Stimulus Category × Age Group	33.66	2	<.001***	0.59	2	.74
Level × Visual Field × Stimulus Category × Age Group	116.81	2	<.001***	1.96	2	.38

*** *p* < .001; **p < .01; *p < .05.

The overall performance for the three stimulus categories in young and older participants indicated there was a significant Stimulus Category × Age Group interaction, both for RT and accuracy. Post hoc pairwise comparisons for RT indicated that: (1) young participants detected letters faster than verbalisable objects and nonverbalisable shapes, and they detected verbalisable objects faster than nonverbalisable shapes (young participants: letters = 490.21 ms, *SE* = 3.55; verbalisable objects = 542.68 ms, *SE* = 3.94; nonverbalisable shapes = 600.75 ms, *SE* = 3.98; *MD_L–VO_* = −51.70 ms, 95% CI [−59.23, −44.18], *z* = −19.69, *p* < .001, *d* = 1.16; *MD_L–NVS_* = −109.66 ms, 95% CI [−119.95, −99.37], *z* = −30.38, *p* < .001, *d* = 2.46; *MD_VO–NVS_* = −57.96 ms, 95% CI [−67.84, −48.07], *z* = −16.71, *p* < .001, *d* = 1.30); (2) older participants detected letters faster than verbalisable objects and nonverbalisable shapes, but there were no RT differences in the detection of verbalisable objects and nonverbalisable shapes (older participants: letters = 568.96 ms, *SE* = 3.63; verbalisable objects = 586.21 ms, *SE* = 3.82; nonverbalisable shapes = 587.07 ms, *SE* = 3.58; *MD_L–VO_* = −15.09 ms, 95% CI [−23.09, −7.09], *z* = −5.38, *p* < .001, *d* = 0.34; *MD_L–NVS_* = −15.67 ms, 95% CI [−24.06, −7.27], *z* = −5.32, *p* < .001, *d* = 0.34; *MD_VO–NVS_* = −0.58 ms, 95% CI [−10.46, 9.31], *z* = −0.17, *p* = .99, *d* = 0.013). Post hoc pairwise comparisons for accuracy indicated that: (1) young participants detected letters more accurately than verbalisable objects and nonverbalisable shapes, and they detected verbalisable objects more accurately than nonverbalisable shapes (young participants: letters = .9970, *SE* = .0014; verbalisable objects = .9860, *SE* = .0036; nonverbalisable shapes = .9420, *SE* = .0115; *MD_L–VO_* = 1.54, 95% CI [0.29, 2.80], *z* = 3.50, *p* = .006, *d*﻿ = 0.88; *MD_L–NVS_* = 3.01, 95% CI [1.67, 4.36], *z* = 6.39, *p* < .001, *d*﻿ = 1.71; *MD_VO–NVS_* = 1.47, 95% CI [0.71, 2.23], *z* = 5.50, *p* < .001, *d*﻿ = 0.84); (2) older participants detected letters more accurately than verbalisable objects and nonverbalisable shapes, but there were no accuracy differences in the detection of verbalisable objects and nonverbalisable shapes (older participants: letters = .9717, *SE* = .0074; verbalisable objects = .8143, *SE* = .0257; nonverbalisable shapes = .7398, *SE* = .0340; *MD_L–VO_* = 2.06, 95% CI [1.44, 2.67], *z* = 9.53, *p* < .001, *d*﻿ = 1.17; *MD_L–NVS_* = 2.49, 95% CI [1.68, 3.30], *z* = 8.75, *p* < .001, *d*﻿ = 1.42; *MD_VO–NVS_* = 0.43, 95% CI [−0.05, 0.91], *z* = 2.58, *p* = .10, *d*﻿ = 0.24).

### Letters

For the stimulus category of letters, there were similar results overall for RT and accuracy. For RT, there were significant main effects of Level, *χ*^2^(1) = 195.78, *p* < .001, and Age Group, *χ*^2^(1) = 95.88, *p* < .001, but there was no significant main effect of Visual Field (*p* = .43). For accuracy, there was a significant main effect of Age Group, *χ*^2^(1) = 8.93, *p* = .003, but there was no significant main effect of Level (*p* = .96) or Visual Field (*p* = .80). Results for all interaction effects relevant to the two research questions for the category of letters are presented below.

#### Right-left hemisphere asymmetry for global-local processing in letters

**Figure 2.**
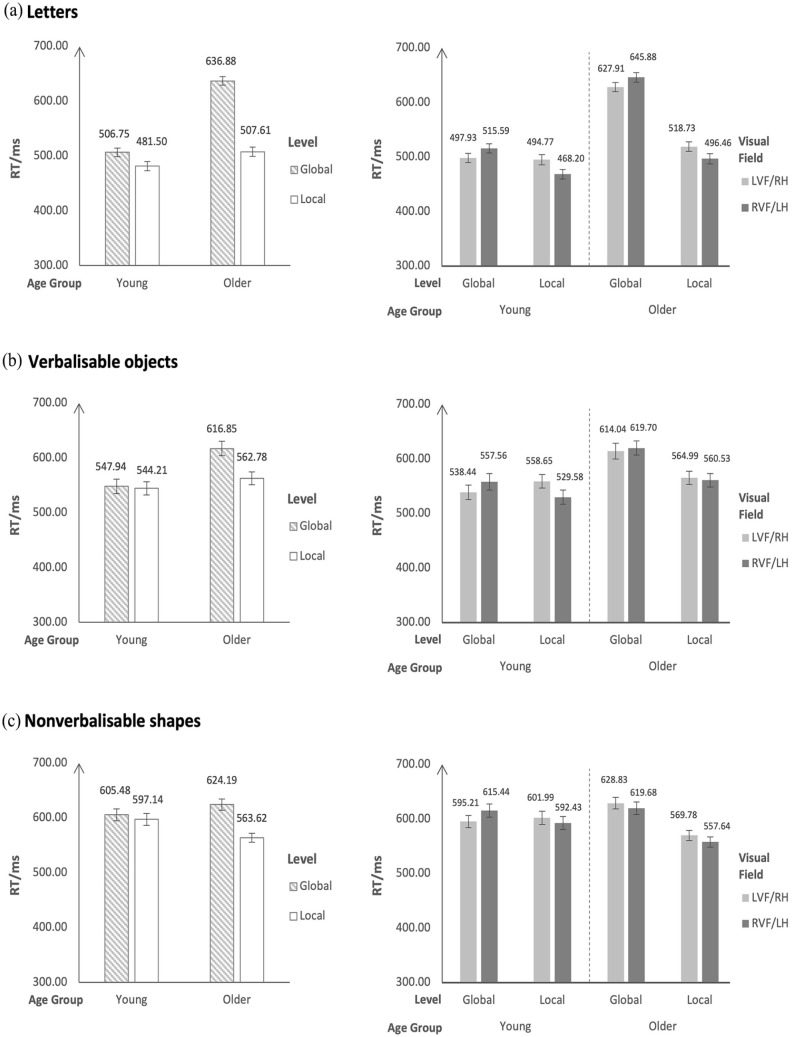
Estimated marginal means and standard errors for the reaction time data. Left panels present the results for Age Group by Level, and right panels present the results for Age Group by Level by Visual Field.

For RT, there was a significant Level × Visual Field interaction, *χ*^2^(1) = 68.92, *p* < .001, but there was no significant Visual Field × Age Group interaction (*p* = .72) nor was there a Level × Visual Field × Age Group interaction (*p* = .56). These results indicated that although there were RT differences in detecting global and local letters in the LVF/RH and RVF/LH, there were no age-group differences in hemispheric asymmetry. Post hoc pairwise comparisons for the Level × Visual Field interaction provided RT evidence for RH specialisation for global processing and LH specialisation for local processing in both young and older participants: global letters were detected faster in the LVF/RH than in the RVF/LH (global: *MD*_LVF/RH–RVF/LH_ = −17.80 ms, 95% CI [−30.80, −4.80], *z* = −3.52, *p* = .003, *d* = 0.31), and local letters were detected faster in the RVF/LH than in the LVF/RH (local: *MD*_LVF/RH–RVF/LH_ = 24.40 ms, 95% CI [12.40, 36.50], *z* = 5.21, *p* < .001, *d* = 0.43). See Figure 2a right panel.

**Figure 3.**
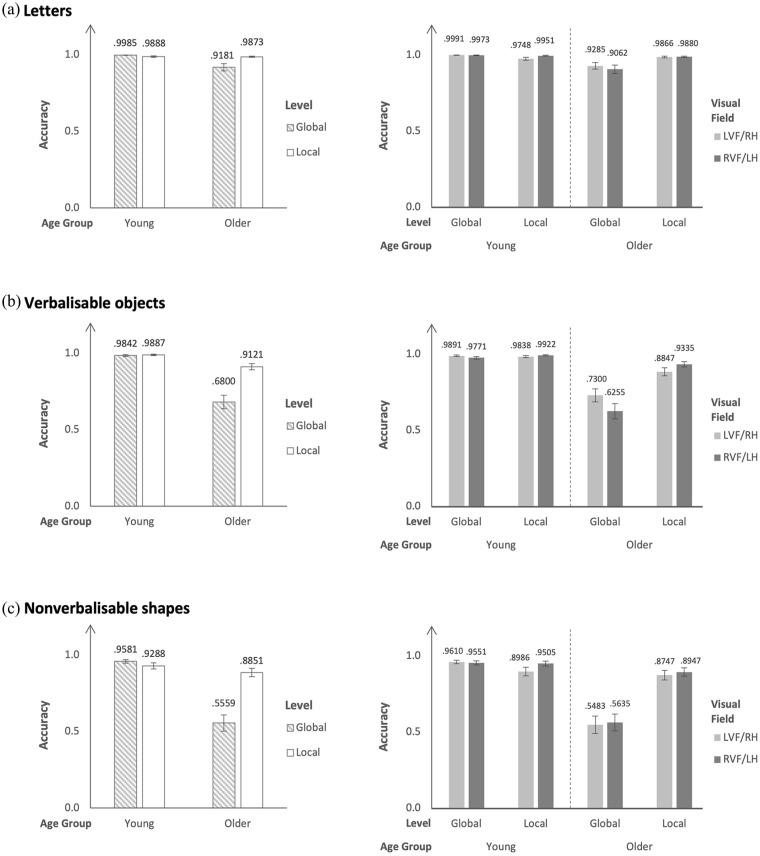
Estimated marginal means and standard errors for the accuracy data. Left panels present the results for Age Group by Level, and right panels present the results for Age Group by Level by Visual Field.

For accuracy, there was a significant Level × Visual Field interaction, *χ*^2^(1) = 5.03, *p* = .02, but there was no significant Visual Field × Age Group interaction (*p* = .60) or Level × Visual Field × Age Group interaction (*p* = .09). None of the post hoc pairwise comparisons for the Level × Visual Field interaction in young and older participants reached significance (all *p*s ⩾ .11). See Figure 3a right panel.

#### Age-related local-processing advantage in letters

For RT, there was a significant Level × Age Group interaction, *χ*^2^(1) = 68.92, *p* < .001. Post hoc pairwise comparisons indicated that: (1) both young participants and older participants were faster in detecting local letters than global letters (young: *MD_global–local_* = 25.28 ms, 95% CI [8.48, 42.10], *z* = 3.87, *p* < .001, *d* = 0.44; older: *MD_global–local_* = 129.30 ms, 95% CI [112.25, 146.30], *z* = 19.48, *p* < .001, *d* = 2.27); (2) both global letters and local letters were detected faster by young participants than by older participants (global: *MD_young–old_* = −130.13 ms, 95% CI [−151.70, 108.59], *z* = −15.52, *p* < .001, *d* = 2.28; local: *MD_young–old_* = −26.11 ms, 95% CI [−49.50, −2.76], *z* = −2.87, *p* = .02, *d* = 0.46). See Figure 2a left panel.

For accuracy, there was a significant Level × Age Group interaction, *χ*^2^(1) = 19.12, *p* < .001. Post hoc pairwise comparisons indicated that: (1) young participants demonstrated no accuracy differences in detecting global and local letters (young: *MD_global–local_* = 1.97, 95% CI [−0.22, 4.19], *z* = 2.31, *p* = .09, *d* = 1.01), whereas older participants were more accurate in detecting local letters than global letters (older: *MD_global–local_* = −1.94, 95% CI [−3.14, −0.73], *z* = −4.14, *p* < .001, *d* = 1.00); (2) global letters were detected more accurately by young participants than by older participants (global: *MD_young–old_* = 4.05, 95% CI [2.06, 6.05], *z* = 5.21, *p* < .001, *d* = 2.08), but there were no accuracy differences between young and older participants in detecting local letters (local: *MD_young–old_* = 0.13, 95% CI [−1.07, 1.33], *z* = 0.28, *p* = .99, *d* = 0.067). See Figure 3a left panel.

### Verbalisable objects

For the stimulus category of verbalisable objects, there were similar results overall for RT and accuracy. For RT, there were significant main effects of Level, *χ*^2^(1) = 9.17, *p* = .002, and Age Group, *χ*^2^(1) = 13.10, *p* < .001, but there was no significant main effect of Visual Field (*p* = .70). For accuracy, there were significant main effects of Level, *χ*^2^(1) = 10.94, *p* < .001, and Age Group, *χ*^2^(1) = 59.91, *p* < .001, but there was no significant main effect of Visual Field (*p* = .63). Results for all interaction effects relevant to the two research questions for the category of verbalisable objects are presented below.

#### Right-left hemisphere asymmetry for global-local processing in verbalisable objects

For RT, there was a significant Level × Visual Field interaction, *χ*^2^(1) = 17.40, *p* < .001, and a significant Level × Visual Field × Age Group interaction, *χ*^2^(1) = 10.89, *p* < .001, but there was no significant Visual Field × Age Group interaction (*p* = .54). These results indicated that although there were RT differences in detecting global and local verbalisable objects in the LVF/RH and RVF/LH, there were also age-group differences in hemispheric asymmetry. Planned additional analyses within each of the two age groups (young, older) were conducted to examine the significant Level × Visual Field × Age Group interaction. In young participants, there was a significant Level × Visual Field interaction, *χ*^2^(1) = 30.54, *p* < .001, whereas in older participants there was no significant Level × Visual Field interaction (*p* = .20). Post hoc pairwise comparisons for the Level × Visual Field interaction provided RT evidence for RH specialisation for global processing and LH specialisation for local processing in young participants only: global verbalisable objects were detected faster in the LVF/RH than in the RVF/LH (global: *MD*_LVF/RH–RVF/LH_ = −20.34 ms, 95% CI [−37.94, −2.73], *z* = −2.97, *p* = .02, *d* = 0.39), and local verbalisable objects were detected faster in the RVF/LH than in the LVF/RH (local: *MD*_LVF/RH–RVF/LH_ = 28.02 ms, 95% CI [11.21, 44.83], *z* = 4.28, *p* < .001, *d* = 0.54). See Figure 2b right panel.

For accuracy, there was a significant Level × Visual Field interaction, *χ*^2^(1) = 14.08, *p* < .001, but there was no significant Visual Field × Age Group interaction (*p* = .85) nor was there a Level × Visual Field × Age Group interaction (*p* = .55). These results indicated that although there were accuracy differences in detecting global and local verbalisable objects in the LVF/RH and RVF/LH there were no age-group differences in hemispheric asymmetry. Post hoc pairwise comparisons for the Level × Visual Field interaction provided accuracy evidence for RH specialisation for global processing and LH specialisation for local processing in both young and older participants: global verbalisable objects were detected more accurately in the LVF/RH than in RVF/LH (global: *MD*_LVF/RH–RVF/LH_ = 0.62, 95% CI [0.04, 1.19], *z* = 2.75, *p* = .03, *d* = 0.37), and local verbalisable objects were detected more accurately in the RVF/LH than in the LVF/RH (local: *MD*_LVF/RH–RVF/LH_ = −0.67, 95% CI [−1.34, −0.004], *z* = −2.58, *p* = .048, *d* = 0.40). See Figure 3b right panel.

#### Age-related local-processing advantage in verbalisable objects

For RT, there was a significant Level × Age Group interaction, *χ*^2^(1) = 23.23, *p* < .001. Post hoc pairwise comparisons indicated that: (1) young participants demonstrated no RT differences in detecting global and local verbalisable objects (young: *MD_global–local_* = 3.89 ms, 95% CI [−25.40, 33.20], *z* = 0.34, *p* = .97, *d* = 0.058), whereas older participants were faster in detecting local verbalisable objects than global verbalisable objects (older: *MD_global–local_* = 54.11 ms, 95% CI [27.50, 80.70], *z* = 5.22, *p* < .001, *d* = 0.81); (2) global verbalisable objects were detected faster by young participants than by older participants (global: *MD_young–old_* = −68.87 ms, 95% CI [−107.00, −30.70], *z* = −4.64, *p* < .001, *d* = 1.03), but there were no RT differences between young and older participants in detecting local verbalisable objects (local: *MD_young–old_* = −18.65 ms, 95% CI [−47.50, 10.20], *z* = −1.66, *p* = .35, *d* = 0.28). See Figure 2b left panel.

For accuracy, there was a significant Level × Age Group interaction, *χ*^2^(1) = 4.60, *p* = .03. Post hoc pairwise comparisons indicated that: (1) young participants demonstrated no accuracy differences in detecting global and local verbalisable objects (young: *MD_global–local_* = −0.35, 95% CI [−1.67, 0.98], *z* = −0.67, *p* = .91, *d* = 0.21), whereas older participants were more accurate in detecting local verbalisable objects than global verbalisable objects (older: *MD_global–local_* = −1.59, 95% CI [−2.35, 0.83], *z* = −5.36, *p* < .001, *d* = 0.95); (2) both global verbalisable objects and local verbalisable objects were detected more accurately by young participants than by older participants (global: *MD_young–old_* = 3.38, 95% CI [2.33, 4.42], *z* = 8.31, *p* < .001, *d* = 2.01; local: *MD_young–old_* = 2.14, 95% CI [0.96, 3.31], *z* = 4.68, *p* < .001, *d* = 1.27). See Figure 3b left panel.

### Nonverbalisable shapes

For the stimulus category of nonverbalisable shapes, with one exception there were similar results overall for RT and accuracy. For RT, there was a significant main effect of Level, *χ*^2^(1) = 17.09, *p* < .001, but there were no significant main effects of Age Group (*p* = .47) or Visual Field (*p* = .66). For accuracy, there were significant main effects of Level, *χ*^2^(1) = 6.09, *p* = .01, Visual Field, *χ*^2^(1) = 4.36, *p* = .04, and Age Group, *χ*^2^(1) = 36.52, *p* < .001. Results for all interaction effects relevant to the two research questions for the category of nonverbalisable shapes are presented below.

#### Right-left hemisphere asymmetry for global-local processing in nonverbalisable shapes

For RT, there was a significant Level × Visual Field interaction, *χ*^2^(1) = 3.91, *p* = .048, and a significant Level × Visual Field × Age Group interaction, *χ*^2^(1) = 5.34, *p* = .02, but there was no Visual Field × Age Group interaction (*p* = .08). These results indicated that although there were RT differences in detecting global and local nonverbalisable shapes in the LVF/RH and RVF/LH, there were also age-group differences in hemispheric asymmetry. Planned additional analyses within each of the two age groups (young, older) were conducted to examine the significant Level × Visual Field × Age Group interaction. In young participants, there was a significant Level × Visual Field interaction, *χ*^2^(1) = 5.51, *p* = .02, whereas in older participants, there was no significant Level × Visual Field interaction (*p* = .93). None of the post hoc pairwise comparisons for the Level × Visual Field interaction in young participants reached significance (all *p*s ⩾ .11). See Figure 2c right panel.

For accuracy, there was a significant Level × Visual Field interaction, *χ*^2^(1) = 6.17, *p* = .01, but there was no significant Visual Field × Age Group interaction (*p* = .38) nor was there a Level × Visual Field × Age Group interaction (*p* = .06). These results indicated that although there were accuracy differences in detecting global and local nonverbalisable shapes in the LVF/RH and RVF/LH, there were no age-group differences in hemispheric asymmetry. Post hoc pairwise comparisons for the Level × Visual Field interaction provided accuracy evidence for LH specialisation for local processing in both young and older participants, but no accuracy evidence for RH specialisation for global processing was found: local nonverbalisable shapes were detected more accurately in the RVF/LH than in the LVF/RH (local: *MD*_LVF/RH–RVF/LH_ = 0.49, 95% CI [0.10, 0.87], *z* = 3.20, *p* = .008, *d* = 0.26), but there were no accuracy differences in detecting global nonverbalisable shapes in the LVF/RH than in the RVF/LH (*p* = .99). See Figure 3c right panel.

#### Age-related local-processing advantage in nonverbalisable shapes

For RT, there was a significant Level × Age Group interaction, *χ*^2^(1) = 45.74, *p* < .001. Post hoc pairwise comparisons indicated that: (1) young participants demonstrated no RT differences in detecting global and local nonverbalisable shapes (young: *MD_global–local_* = −8.11 ms, 95% CI [−32.44, 16.20], *z* = −0.86, *p* = .83, *d* = 0.11), whereas older participants were faster in detecting local nonverbalisable shapes than global nonverbalisable shapes (older: *MD_global–local_* = 60.55 ms, 95% CI [37.80, 83.29], *z* = 6.84, *p* < .001, *d* = 0.82); (2) there were no RT differences between young and older participants in detecting global nonverbalisable shapes (global: *MD_young–old_* = 18.94 ms, 95% CI [−10.2, 48.07], *z* = 1.67, *p* = .34, *d* = 0.26), but local nonverbalisable shapes were detected faster by older participants than by young participants (local: *MD_young–old_* = 33.50 ms, 95% CI [7.64, 59.40], *z* = 3.33, *p* < .001, *d* = 0.45). See Figure 2c left panel.

For accuracy, there was a significant Level × Age Group interaction, *χ*^2^(1) = 22.57, *p* < .001. Post hoc pairwise comparisons indicated that: (1) young participants demonstrated no accuracy differences in detecting global and local nonverbalisable shapes (young: *MD_global–local_* = 0.56, 95% CI [−0.44, 1.56], *z* = 1.45, *p* = .47, *d* = 0.30), whereas older participants were more accurate in detecting local nonverbalisable shapes than global nonverbalisable shapes (older: *MD_global–local_* = −1.82, 95% CI [−2.65, −0.99], *z* = −5.64, *p* < .001, *d* = 0.98); (2) global nonverbalisable shapes were detected more accurately by young participants than by older participants (global: *MD_young–old_* = 2.91, 95% CI [1.99, 3.82], *z* = 8.18, *p* < .001, *d* = 1.06), but there was no accuracy differences between young and older participants in detecting local nonverbalisable shapes (local: *MD_young–old_* = 0.53, 95% CI [−0.50, 1.56], *z* = 1.32, *p* = .55, *d* = 0.29). See Figure 3c left panel.

### Summary of results

#### Right-left hemisphere asymmetry for global-local processing

Overall, our findings indicated that there was support (RT and/or accuracy) for hemispheric asymmetry for global-local processing (RH-global specialisation and LH-local specialisation) in both young and older participants. For letters, both young and older participants were significantly faster in detecting global letters in the LVF/RH compared to the RVF/LH, and in detecting local letters in the RVF/LH compared to the LVF/RH. There was no support in the accuracy data for hemispheric asymmetry for letters. For verbalisable objects, both young and older participants were significantly more accurate in responding to global verbalisable objects in the LVF/RH compared to the RVF/LH, and in responding to local verbalisable objects in the RVF/LH compared to the LVF/RH. But only young participants were significantly faster in detecting global verbalisable objects in the LVF/RH compared to the RVF/LH, and in detecting local verbalisable objects in the RVF/LH compared to the LVF/RH. In contrast to the significant findings for hemispheric asymmetry in letters and verbalisable objects, the findings for nonverbalisable shapes indicated support only for LH-local specialisation: both young and older participants were significantly more accurate in responding to local nonverbalisable shapes in the RVF/LH compared to the LVF/RH. There was no support in the RT or accuracy data for RH-global specialisation for nonverbalisable shapes.

#### Age-related local-processing advantage

Overall, the within-group comparisons indicated that there was support for a local-processing advantage in older participants, in that older participants were significantly faster and more accurate in detecting local-level stimuli compared to global-level stimuli across all three stimulus categories. In contrast, young participants—with one exception—demonstrated no RT or accuracy differences in detecting global-level and local-level stimuli. The one exception was for letters, where young participants were faster in detecting the local letters compared to the global letters.

Overall, the between-group comparisons for local-level stimuli indicated that—with three exceptions—there were no RT or accuracy differences found between the two age groups. The three exceptions were as follows: young participants were faster in detecting local letters compared to older participants; young participants were more accurate in detecting local verbalisable objects compared to older participants; older participants were faster in detecting local nonverbalisable shapes compared to young participants. The between-group comparisons for global-level stimuli indicated that—with one exception—young participants were faster and/or more accurate compared to older participants. The one exception was for global nonverbalisable shapes where there were no RT differences between the two age groups.

## Discussion

In the present study, we examined hemispheric asymmetry for global-local processing in young and older participants with hierarchical figures from three different stimulus categories (letters, verbalisable objects, and nonverbalisable shapes). For letters and verbalisable objects, we found that regardless of stimulus category, young and older participants demonstrated RH-global specialisation (i.e., global-level stimuli were detected faster and/or more accurately in the LVF/RH compared to the RVF/LH) and LH-local specialisation (i.e., local-level stimuli were detected faster and/or more accurately in the RVF/LH compared to the LVF/RH). For nonverbalisable shapes, young and older participants demonstrated LH-local specialisation but we found no evidence for RH-global specialisation.

For older participants, in addition to finding RH-global specialisation and LH-local specialisation for letters and verbalisable objects, we found a local-processing advantage (i.e., local-level stimuli detected faster and/or more accurately compared to global-level stimuli) for all three stimulus categories (letters, verbalisable objects, and nonverbalisable shapes). First, our results support findings from previous studies (e.g., Insch et al., 2012; Lux et al., 2008; Oken et al., 1999; Slavin et al., 2002) that have compared older adults’ own performance differences in global-local processing (i.e., within-group comparisons), and found a local-processing advantage. Second, our results extend previous findings because we compared the older participants’ performance with that of young participants (i.e., between-group comparisons). Older participants were slower and/or less accurate when responding to global-level stimuli but their responses to local-level stimuli were comparable to that of young participants. Furthermore, to the best of our knowledge, this is the first demonstration that, across three different stimulus categories, older participants’ slower and/or less accurate global-processing performance was not specific to stimuli presented in the LVF/RH.

### Effects of stimulus category on hemispheric asymmetry for global-local processing

Our experiment setup allowed us to investigate two competing accounts of hemispheric asymmetry in global-local processing—the stimulus-independent account and the stimulus-dependent account. The s*timulus-independent account* suggests that the stimulus category does not influence hemispheric asymmetry (e.g., Brederoo et al., 2017), whereas the *stimulus-dependent account* suggests that hemispheric asymmetry is influenced by the verbal-visuospatial processing demands associated with the stimulus category (e.g., Fink, Marshall, et al., 1997; Kéïta & Bedoin, 2011).

#### Letters and verbalisable objects

In young participants, our findings for letters and verbalisable objects demonstrated the predicted pattern of RH-global specialisation and LH-local specialisation. These findings are in line with previous behavioural studies reporting RH-global specialisation and LH-local specialisation in young adults: for letters (e.g., RT: Hübner, 1998; Yovel et al., 2001); for verbalisable objects (e.g., RT: Oken et al., 1999; RT and accuracy: Peterson & Peterson, 2014); and for both letters and verbalisable objects (accuracy: Brederoo et al., 2017). Our findings for letters are also in line with the neuroimaging data from Fink, Halligan, et al. (1997) and Fink, Marshall, et al. (1997) and the behavioural data from Kéïta and Bedoin (2011). But our findings for verbalisable objects did not replicate these researchers’ findings of a reversed pattern of hemispheric asymmetry (i.e., RH-*local* specialisation and LH-*global* specialisation). Brederoo et al. (2017) have argued that the reversal of hemispheric asymmetry for verbalisable objects in the study by Fink, Marshall, et al. (1997) and by Kéïta and Bedoin (2011) may have been a result of two confounding factors—greater visual complexity of the hierarchical verbalisable objects compared to that of the hierarchical letters, and the greater number of local-level stimuli used in the hierarchical objects. In the present study, we did not observe a reversal of hemispheric asymmetry for verbalisable objects even though we believe our hierarchical verbalisable objects were visually more complex than our hierarchical letters. Therefore, we argue that the reversal of hemispheric asymmetry for verbalisable objects may more likely relate to the number of local-level stimuli used in previous studies.

Brederoo et al.’s (2017) hierarchical letter was composed of seven to 13 local-level stimuli, and the hierarchical verbalisable object was composed of eight to 12 local-level stimuli. In contrast, in the two studies by Fink, Halligan, et al. (1997) and Fink, Marshall, et al. (1997), the hierarchical letter was composed of 11 to 18 local-level stimuli but the hierarchical verbalisable object was composed of 47 to 49 local-level stimuli. Similarly, in the study by Kéïta and Bedoin (2011), the hierarchical letter was composed of 16 to 26 local-level stimuli but the hierarchical verbalisable object was composed of 24 to 32 local-level stimuli (also see Table 1). In the present study, our hierarchical letter was composed of seven to 10 local-level stimuli, and our hierarchical verbalisable object was composed of 10 to 15 local-level stimuli. Perhaps more notable is that the number of local-level stimuli (10 to 15) in our hierarchical verbalisable object was similar to the number of local-level stimuli (8 to 12) in the study by Brederoo et al., who also did not find a reversal of hemispheric asymmetry. As explained by Brederoo et al., hierarchical figures with a large number of local-level stimuli may lead to the reversal of hemispheric asymmetry because large numbers of local-level stimuli may result in local-level stimuli being perceived as the textural background that makes up the global-level stimulus, rather than as distinct individual components. These effects would reduce the reliability of finding hemispheric asymmetry for global-local processing (Kimchi & Merhav, 1991).

In older participants, as in young participants, our findings for letters and verbalisable objects demonstrated the predicted pattern of RH-global specialisation and LH-local specialisation. Our findings with verbalisable objects are in line with Oken et al. (1999), who used verbalisable objects as stimuli and conducted the only other previous behavioural study with a divided visual field presentation to examine hemispheric asymmetry for global-local processing in older adults. Our findings with letters extend the findings from Oken et al. with their verbalisable objects, and confirm that older adults demonstrate RH-global specialisation and LH-local specialisation for both letters and verbalisable objects.

#### Nonverbalisable shapes

In both young and older participants, our accuracy data for nonverbalisable shapes demonstrated LH-local specialisation but there was no evidence (neither for RT nor accuracy) found for RH-global specialisation (i.e., there were no performance differences in detecting global-level nonverbalisable shapes presented in the LVF/RH compared to those presented in the RVF/LH). Martinez et al. (1997) conducted the only previous behavioural study examining hemispheric asymmetry for global-local processing of nonverbalisable shapes, and like us they used a divided visual field presentation. In young adults, they found LH-local specialisation in the absence of RH-global specialisation for nonverbalisable shapes. One possible explanation for these findings with nonverbalisable shapes may be the difficulty of the stimulus category. Nonverbalisable shapes are unfamiliar novel shape patterns, which are processed less automatically than letters or verbalisable objects. Importantly, although Martinez et al. found RT evidence for LH-local specialisation of nonverbalisable shapes, their neuroimaging findings demonstrated evidence not only of greater LH activation for local processing (LH-local specialisation) but also of greater RH activation for global processing (RH-global specialisation). This inconsistency between their RT and neuroimaging findings for hemispheric asymmetry also suggests that behavioural measures may not be sensitive enough to detect RH-global specialisation for nonverbalisable shapes.

In young participants, our findings indicated longer RT for nonverbalisable shapes compared to letters and verbalisable objects, and the RT for nonverbalisable shapes were longer than those reported by Martinez et al. (1997).^8^ In the present study, the longer RT for nonverbalisable shapes may reflect overall greater processing time, thus making it difficult to isolate the specific effects of each hemisphere for processing global-local information. Importantly, the longer RT for nonverbalisable shapes were also consistently found in our pilot study (see Footnote 5), where young participants were slower at detecting nonverbalisable shapes compared to verbalisable objects, regardless of whether they performed the hierarchical nonverbalisable shapes experiment first and the hierarchical verbalisable objects experiment second or in the reverse order. One factor that may have contributed to the longer RT in the present study could be the greater task difficulty involved in the divided-attention paradigm compared to the focused-attention paradigm used by Martinez et al. It has been suggested (see Müller-Oehring et al., 2007) that a divided-attention paradigm poses greater task difficulty because it requires concurrent allocation of attention to both the global level and the local level of the stimulus, in contrast to the allocation of attention to only one processing level in a focused-attention paradigm. We adopted a divided-attention paradigm because Yovel et al. (2001) reported that a divided-attention (as compared to a focused-attention) paradigm produces a greater effect size for hemispheric asymmetry for global-local processing. However, the findings from Yovel et al. were based on young adults’ performance with letters, and they did not test the effects of other stimulus categories, or the effects of ageing. Another factor that may have contributed to our longer RT for nonverbalisable shapes, compared to those of Martinez et al., is that we had two nonverbalisable-shape targets whereas they had only one. We suggest that the greater number of targets in the present study increased the difficulty of our task compared to that of Martinez et al.’s study, and this difference in task difficulty was reflected in the participants’ RT performance.

Considering the limited amount of research on hemispheric asymmetry for global-local processing of nonverbalisable shapes, future research is needed to examine systematically the effects of task difficulty (e.g., a divided- *versus* focused-attention paradigm; number of targets requiring a response in the experiment) on finding RH-global specialisation and LH-local specialisation in different age groups.

### Age-related local-processing advantage

Our experiment setup also allowed us to investigate the extent to which the right-hemisphere ageing hypothesis (i.e., the RH is more vulnerable to ageing than the LH) can explain previous findings of a local-processing advantage in older adults (i.e., local-level stimuli detected faster and/or more accurately compared to global-level stimuli).

Given the proposal that the RH is more vulnerable to ageing than the LH (Albert, 1981; Brown & Jaffe, 1975; Klisz, 1978), we sought to determine whether the local-processing advantage we found in older participants could be attributed to age-related changes in RH functioning. Our within-group analyses revealed that older participants were faster and more accurate in detecting local-level stimuli compared to global-level stimuli for all three stimulus categories (letters, verbalisable objects, and nonverbalisable shapes). These findings are in line with previous behavioural studies reporting a local-processing advantage in older adults: for letters (e.g., RT and accuracy: Lux et al., 2008); for numbers (e.g., RT: Slavin et al., 2002); and for verbalisable objects (e.g., RT: Oken et al., 1999; accuracy: Insch et al., 2012). Our between-group analyses revealed that older participants were slower and less accurate in detecting global-level stimuli compared to young participants but there were no age-group differences in detecting local-level stimuli. The local-processing advantage in older adults reported in previous studies was based on performance differences between global and local processing in the data of the older adults (i.e., within-subject comparisons, as there was no report of age-group differences). Our findings confirm that older participants demonstrate a local-processing advantage regardless of stimulus category and that this local-processing advantage is driven not by better performance in local processing but by worse performance in global processing.

Oken et al. (1999) tested young and older participants with verbalisable objects using a divided visual field presentation. In addition to finding a local-processing advantage in older adults, Oken et al. also reported that older participants did not demonstrate age-related changes in overall performance in the LVF/RH as compared to the RVF/LH. They suggested that their findings did not support the right-hemisphere ageing hypothesis. They further argued that if the local-processing advantage in older adults was associated with the RH ageing process then the older adults’ RT performance for stimuli presented in the LVF/RH should be worse, regardless of the global-local processing level. We found support for Oken et al.’s proposal since our older participants demonstrated a local-processing advantage for all three stimulus categories. Importantly, their overall RT and accuracy performance for stimuli presented in the LVF/RH was comparable to their performance in the RVF/LH, regardless of whether the stimuli were global stimuli or local stimuli. These findings suggest that there are age-related differences in global processing, but that these age-related differences are evident for stimuli presented in the LVF/RH and in the RVF/LH.

Two alternative explanations have been put forward to explain the local-processing advantage observed in older adults—narrowed attention and difficulties with visual processing at greater eccentricities. The first explanation suggests that the local-processing advantage in older adults may be a compensatory mechanism for addressing poorer visual input from age-related deterioration in optical structures (Kosslyn et al., 1999; see Oken et al., 1999). If attention is narrowed with age, older adults will demonstrate better performance for local processing compared to young adults. However, our findings did not support this, as we observed that the local-processing advantage in older participants was driven not by faster and more accurate performance for local processing but instead by slower and less accurate performance for global processing. The second explanation suggests that the local-processing advantage in older adults may relate to difficulties with visual processing at greater eccentricities (Cerella, 1985; Hartley & McKenzie, 1991). Oken et al. (1999) suggested that in a divided visual field presentation, the average position of the global-level stimulus is farther from central fixation (i.e., at a greater eccentricity) compared to the local-level stimuli that are closer to central fixation. One limitation to be considered with this proposal is that previous studies reporting a local-processing advantage in older adults have also presented their stimuli centrally (e.g., Lux et al., 2008; Slavin et al., 2002), in which the position of the global stimulus was at central fixation.

Our behavioural findings of age-related differences for global processing in older participants are consistent with the electrophysiological study conducted by Lithfous et al. (2016). They reported behavioural and ERP (event-related potentials) data for the processing of centrally-presented hierarchical letters in young and older participants. Their RT data confirmed an age-related local-processing advantage, in which older adults were faster at detecting local-level letters compared to global-level letters. Their ERP data indicated that, while young and older adults demonstrated the same amplitudes for the early N2 component (~250 ms–340 ms), older adults demonstrated reduced amplitudes for the later P300 component (~340 ms–480 ms). The P300 component has been suggested to relate to perceptual grouping of local-level stimuli for the identification of the global-level stimulus in hierarchical figures, and this occurs at a late stage of global-local processing (Dalrymple et al., 2009). The findings from Lithfous et al. suggest that the local-processing advantage in older adults may be driven by age-related changes in global processing that are associated with the P300 component. To the best of our knowledge, no ERP study to date has investigated global-local processing in older adults in the context of hemispheric asymmetry. Future research could consider incorporating ERP data with behavioural measures of hemispheric asymmetry (i.e., a divided visual field presentation) to investigate whether age-related changes in RH functioning can be observed specifically for the P300 component.

## Conclusion

The present study is the first to examine hemispheric asymmetry for global-local processing in young and older participants with hierarchical figures from three different stimulus categories (letters, verbalisable objects, and nonverbalisable shapes) in a within-subjects design using a divided-attention paradigm and divided visual field unilateral presentation. For letters and verbalisable objects, our findings in both age groups support the *stimulus-independent* account (e.g., Brederoo et al., 2017) and do not support the stimulus-dependent account (e.g., Fink, Halligan, et al., 1997; Fink, Marshall, et al., 1997). For letters and verbalisable objects, our findings in young and older participants indicate that hemispheric asymmetry for global-local processing demonstrates the same pattern of RH-global specialisation and LH-local specialisation regardless of stimulus category. However, for nonverbalisable objects, our findings in young and older participants reveal LH-local specialisation in the absence of RH-global specialisation. Further investigation is warranted to determine whether this finding can be attributed to the greater task difficulty of the nonverbalisable shapes. Our findings in older participants confirmed there was a local-processing advantage across all three stimulus categories (letters, verbalisable objects, and nonverbalisable shapes), and that this local-processing advantage was driven not by better performance in local processing but by worse performance in global processing. Importantly, the findings indicate that the age-related differences found were not specific to the LVF/RH when we compared the older adults’ own performance in the LVF/RH with that in the RVF/LH. Overall, our findings challenge the right-hemisphere ageing hypothesis, prompting the need for future research to investigate the mechanisms underlying these findings for global processing in older adults.

## Notes

1. For unilaterally-presented stimuli (i.e., stimuli presented either to the LVF or to the RVF), the local-processing advantage for behavioural measures is calculated by comparing the participants’ performance for processing global-level and local-level stimuli after collapsing (i.e., averaging) across visual field of presentation.

2. Bruyer and Scailquin (2000) found a global-processing advantage for both RT and accuracy differences but they did not report the number of local-level stimuli used in their hierarchical figures.

3. The main research question concerned hemispheric asymmetry, and thus the power analysis was based on the RT effect size for the Level (global, local) × Visual Field (LVF, RVF) interaction for each of the two age groups. In young participants, an effect size of η^p^^2^ = .40 was adopted based on the findings from Yovel et al. (2001; see p. 1381), who had an effect size of η^p^^2^ = .59 for letters, and from Martinez et al. (1997; see p. 1688), who had an effect size of η^p^^2^ = .40 for nonverbalisable shapes (as retrieved from the F statistics they reported). We opted for the smaller effect size of η^p^^2^  = .40 from Martinez et al., as it required a larger minimum sample size to achieve the same level of power compared to the larger effect size of η^p^^2^  = .59 from Yovel et al. In older participants, the effect size of η^p^^2^  = .28 was adopted based on the effect size retrieved from the F statistics reported by Oken et al. (1999; see p. 79), which was the only previous study examining hemispheric asymmetry in older adults using verbalisable objects.

4. The eccentricity of 0.50° for the presentation of the hierarchical figures was based on the study by Yovel et al. (2001). Visual acuity decreases as eccentricity increases from central fixation (Lindell & Nicholls, 2003), and the RH is specialised for processing stimuli with low visual acuity (Christman, 1989; Sergent, 1983). Yovel et al. suggested that stimuli with low visual acuity due to a large eccentricity may inadvertently enhance RH activity, introducing a bias in hemispheric asymmetry for global-local processing.

5. To ensure that experiment order would not influence overall performance on nonverbalisable shapes and verbalisable objects, we conducted a pilot study with 18 young participants, who self-reported being of European descent: 10 participants performed the nonverbalisable shapes experiment first and the verbalisable objects experiment second; 8 participants performed the verbalisable objects experiment first and the nonverbalisable shapes experiment second. Our RT data revealed a significant main effect for Stimulus Category (nonverbalisable shapes, verbalisable objects), χ^2^(1) = 261.98, *p* < .001), but there was no significant main effect for Experiment Order (Order 1, Order 2), *p* = .11 nor was there a significant effect for the Stimulus Category × Experiment Order interaction, *p* = .23. The significant main effect of stimulus category indicated that, regardless of experiment order, participants were significantly slower at detecting nonverbalisable shapes (668.70 ms) compared to verbalisable objects (587.23 ms).

6. Reporting both RT and accuracy allowed for direct comparisons to be made between our findings and previous research on ageing and global-local processing. The majority of studies we have cited examining ageing and global-local processing have reported both RT and accuracy analyses (see Agnew et al., 2016; Bruyer et al., 2003; Bruyer & Scailquin, 2000; Georgiou-Karistianis et al., 2006; Lithfous et al., 2016; Lux et al., 2008; Oken et al., 1999; Roux & Ceccaldi, 2001; Slavin et al., 2002; Staudinger et al., 2011).

7. To the best of our knowledge, there is currently no published study or R package available for computing effect size in generalised mixed-effects models (e.g., gamma and logistic mixed-effects models) with an ANOVA-style interpretation (i.e., main effects, interaction effects, and post hoc pairwise comparisons). The effect size *d* reported in this study was designed for calculating the effect size of regression coefficients in linear mixed-effects models (Brysbaert & Stevens, 2018; Westfall et al., 2014).

8. Martinez et al. (1997) reported RT data for the Level × Visual Field interaction in young participants: global nonverbalisable shapes in the LVF/RH = 544 ms; global nonverbalisable shapes in the RVF/LH = not reported; local nonverbalisable shapes in the LVF/RH = 586 ms; local nonverbalisable shapes in the RVF/LH = 551 ms (see p. 1688).
